# Intramuscular Evaluation of Chimeric Locked Nucleic Acid/2′*O*Methyl-Modified Antisense Oligonucleotides for Targeted Exon 23 Skipping in Mdx Mice

**DOI:** 10.3390/ph14111113

**Published:** 2021-10-30

**Authors:** Michaella Georgiadou, Melina Christou, Kleitos Sokratous, Jesper Wengel, Kyriaki Michailidou, Kyriacos Kyriacou, Andrie Koutsoulidou, Nikolaos P. Mastroyiannopoulos, Leonidas A. Phylactou

**Affiliations:** 1 Molecular Genetics, Function & Therapy Department, The Cyprus Institute of Neurology & Genetics, Nicosia 2371, Cyprus; m.georgiadou@nipd.com (M.G.); christoum@cing.ac.cy (M.C.); andriek@cing.ac.cy (A.K.); chiefscientist@research.org.cy (N.P.M.); 2 Bioinformatics Group, The Cyprus Institute of Neurology & Genetics, Nicosia 2371, Cyprus; kleitos.sokratous@omass.com; 3 Department of Physics, Chemistry and Pharmacy, University of Southern Denmark, DK-5230 Odense, Denmark; jwe@sdu.dk; 4 Biostatistics Unit, The Cyprus Institute of Neurology & Genetics, Nicosia 2371, Cyprus; kyriakimi@cing.ac.cy; 5 Cancer Genetics, Therapeutics & Ultrastructural Pathology Department, The Cyprus Institute of Neurology & Genetics, Nicosia 2371, Cyprus; kyriacos@cing.ac.cy

**Keywords:** DMD, exon skipping, antisense oligonucleotides, LNA/2′*O*Me, mdx

## Abstract

Duchenne muscular dystrophy (DMD) is a fatal disorder characterised by progressive muscle wasting. It is caused by mutations in the dystrophin gene, which disrupt the open reading frame leading to the loss of functional dystrophin protein in muscle fibres. Antisense oligonucleotide (AON)-mediated skipping of the mutated exon, which allows production of a truncated but partially functional dystrophin protein, has been at the forefront of DMD therapeutic research for over two decades. Nonetheless, novel nucleic acid modifications and AON designs are continuously being developed to improve the clinical benefit profile of current drugs in the DMD pipeline. We herein designed a series of 15mer and 20mer AONs, consisting of 2′*O*-Methyl (2′*O*Me)- and locked nucleic acid (LNA)-modified nucleotides in different percentage compositions, and assessed their efficiency in inducing exon 23 skipping and dystrophin restoration in locally injected muscles of mdx mice. We demonstrate that LNA/2′*O*Me AONs with a 30% LNA composition were significantly more potent in inducing exon skipping and dystrophin restoration in treated mdx muscles, compared to a previously tested 2′*O*Me AON and LNA/2′*O*Me chimeras with lower or higher LNA compositions. These results underscore the therapeutic potential of LNA/2′*O*Me AONs, paving the way for further experimentation to evaluate their benefit-toxicity profile following systemic delivery.

## 1. Introduction

Duchenne Muscular Dystrophy (DMD) is an X-linked recessive disorder characterised by progressive muscle wasting, loss of ambulation in early adolescence and premature mortality due to cardiorespiratory complications. The disorder is mainly caused by nonsense or frame-shift mutations in the dystrophin gene, which disrupt the open reading frame, leading to lack of functional dystrophin protein in the sarcolemma of muscle fibres [1,2]. A milder variant of DMD is Becker Muscular Dystrophy (BMD), caused by deletions in the dystrophin gene that maintain the open reading frame, resulting in the production of a shorter but partially functional dystrophin protein [3]. This variation in the genetic etiology and severity of the two disorders inspired the development of a powerful therapeutic tool, the antisense oligonucleotides (AONs), which induce the skipping of the mutated exon changing the deletion from out-of-frame (severe DMD phenotype) to in-frame (mild BMD phenotype) [4,5,6]. Theoretically, AON-mediated exon skipping is applicable to at least 70% of DMD patients, where the skipping of a single exon is required, rising up to 90% if multiexon skipping is achieved. An important challenge lies in the fact that AONs are mutation specific, which means that different AON sequences need to be designed in order to target the numerous DMD mutations that have been identified. Nonetheless, mutations clustered in exons 43 to 55 can be strategically targeted using multiexon skipping to benefit over 50% of DMD patients, while AON-induced skipping of exon 51 alone can benefit 13–14% of DMD individuals [7,8].

Over the last two decades, a substantial number of chemically modified AONs have been developed for DMD therapy with encouraging results. AON chemistries include modifications to the phosphodiester (PO) backbone (e.g., phosphorothioate and phosphorodiamidate morpholino) and/or the sugar ring (e.g., 2′-*O*-Methyl, locked nucleic acid and tricyclo-DNA) and aim at increasing binding affinity to the target sequence, as well as resistance to nuclease degradation in the serum and cytoplasm. One of the first two AONs to be tested in clinical trials for DMD therapy was based on the phosphorodiamidate morpholino (PMO) chemistry, which consists of charge neutral phosphorodiamidate linkages and a morpholine moiety in the place of the sugar ribose. Because of their non-ionic nature, PMO AONs show increased tissue accumulation in vivo as they do not interact undesirably with cellular components [9]. After a 3.5-year clinical assessment, the PMO oligomer targeting exon 51 (Eteplirsen, brand name: Exondys 51) demonstrated a 0.9% increase in dystrophin levels and a slower decline in ambulation in treated individuals compared to historical controls [10,11,12,13]. Based on these results, Eteplirsen was granted accelerated FDA approval making it the first drug to be approved for DMD therapy in the U.S. [14,15,16]. Subsequent clinical trials have shown that long-term treatment with Eteplirsen attenuates respiratory decline and stabilizes cardiac and upper limb function in both patients who remain ambulatory and patients who lose ambulation during the course of the treatment [17,18]. Three more PMO-based drugs have subsequently been approved in the U.S. for DMD treatment (under the accelerated approval program), offered to patients amenable to exon 45 (Casimersen, brand name: Amondys 45) or exon 53 (Golodirsen, brand name: Vyondys 53 and Viltolarsen, brand name: Viltepso) skipping [19,20,21,22,23,24,25].

The second exon 51-targeting AON to be tested in clinical trials consisted of 2′-*O*-Methyl (2′*O*Me)-modified nucleosides on a negatively charged phosphorothioate (PS)-modified backbone. Despite promising pre-clinical data, long-term subcutaneous delivery of the 2′*O*MePS AON drisapersen in DMD patients showed minimal to no functional benefit and caused adverse injection-site and systemic reactions [26,27,28,29]. Consequently, the U.S. Food and Drug Administration (FDA) denied drug approval. AONs with phosphorothioate (PS) backbones have long been reported to show dose-dependent side-effects in vivo, inferring that the side-effects seen after drisapersen treatment in DMD patients may be caused by the PS modification [30]. Despite increasing AON stability, PS linkages reduce the binding affinity of an oligonucleotide towards its target sequence, leading to a decrease in AON specificity. Indeed, in vitro studies have shown that PS AONs associate with key cellular proteins in a sequence-independent manner, resulting in nucleolar stress, p53 activation and apoptotic cell death [31,32,33,34,35,36].

Recently developed AONs for DMD therapy are based on the third-generation sugar modifications tricyclo-DNA (tcDNA) and locked nucleic acid (LNA), which demonstrate remarkably higher thermodynamic stability (binding affinity) compared to second-generation 2′-*O*-alkyl chemistries and increased resistance to nuclease degradation. Systemic delivery of a tcDNA/PS AON in mdx mice resulted in high restoration of dystrophin expression in skeletal muscle, heart and diaphragm tissues, and to a lower extent in the brain, leading to both functional and neurobehavioral improvement. Notably, tcDNA/PS was more efficient than the PMO and 2′*O*MePS AONs and demonstrated an encouraging safety profile with minimal histological changes and slight differences in serum and urinary levels of renal toxicity biomarkers [37,38].

LNA nucleoside analogues consist of a methylene bridge joining the 2′-oxygen to the 4′-carbon, resulting in a locked C3′-endo conformation that closely resembles A-form RNA. This conformational feature essentially pre-organizes LNA-modified AONs for RNA binding [39]. LNA nucleotides impart impressive thermodynamic stability, the highest among all other chemistries, increasing the Tm of a duplex by approximately 5.6 °C per insert [40]. Though much more potent, LNA-modified AONs are more tolerant to mismatches increasing the risk of unintended, off-target interactions. In a comparative study of chemically modified AONs targeting human exon 46, a 14mer all-LNA AON induced the highest levels of exon skipping in both unaffected control- (85%) and patient-derived (98%) myotubes. By contrast, a 20mer 2′*O*Me/PS AON was remarkably more efficient in the DMD versus control myotubes (75 versus 20%, respectively), a finding that questioned the sequence specificity of LNA versus 2′*O*Me AONs. Indeed, while demonstrating higher skipping efficiencies, LNA AONs containing up to three mismatches showed much less sequence specificity compared to mismatched 2′*O*Me AONs [41]. Consequently, recent studies have focused on addressing the optimal design of splice-switching LNA AONs, by altering the number of incorporated LNAs as well as the length of the oligomer sequence [42,43]. A 16mer LNA/DNA AON with an LNA incorporation of 60% efficiently induced skipping of exon 51 and restored dystrophin expression in the plasma membrane of patient-derived myotubes, thus highlighting the value of short LNA AONs for splice-switching therapy [43].

Attempting to limit sequence-dependent and sequence-independent off-target interactions, associated with the LNA and PS modifications, respectively, Le and colleagues designed short mixmer AONs incorporating a combination of 2′*O*Me and LNA nucleotides on a full PS backbone. The introduction of four LNAs in a 14mer 2′*O*Me/PS AON, showing no exon skipping activity, significantly improved the yield of exon 23-skipped RNA in cultured H2K mdx mouse myotubes, underscoring the value of short LNA/2′*O*Me AONs for DMD therapy [44]. Recently, chimeric LNA/2′*O*Me AONs (14–18mer) have also been evaluated for Myotonic Dystrophy type 1 (DM1) therapy, demonstrating strong steric-blocking activities in cultured human DM1 myoblasts and in locally injected muscles of DM1 mice [45]. To further evaluate the optimal length and percentage chemistry composition of LNA/2′*O*Me chimeras for DMD therapy, we herein designed a series of 20mer LNA/2′*O*Me AONs by introducing an increasing number of LNA nucleotides to a previously published 20mer 2′*O*MePS sequence targeting exon 23 of the mouse dystrophin pre-mRNA [46,47]. Truncated (15mer) sequence analogues with similar LNA compositions were also created and compared to a corresponding 2′*O*Me AON. The AONs were delivered intramuscularly into the tibialis anterior (TA) muscle of mdx mice and evaluated for targeted exon 23 skipping and rescued dystrophin protein expression. This study represents the first in vivo evaluation of the LNA/2′*O*Me AON design for DMD therapy.

## 2. Results

### 2.1. Evaluation of Exon Skipping Efficiency of Chimeric LNA/2′OMe AONs after Intramuscular Delivery in Mdx Mice

In order to address the optimal design of chimeric LNA/2′OMe AONs for exon skipping activity in vivo, we created a series of 15mer (sLNA-3, sLNA-5 and sLNA-6) and 20mer (LNA-4, LNA-6 and LNA-8) LNA/2′OMe oligomers exhibiting an LNA incorporation of 20, 30 and 40% respectively, for each group, and compared their efficiencies to a 15mer and 20mer 2′OMe-modified AON. Both 2′OMe and chimeric LNA/2′OMe AONs were synthesized on a fully modified PS backbone. An equimolar dose of each AON was injected into the TA muscle of 8-week-old mdx mice and evaluated for exon 23 skipping by RT-PCR, two weeks after treatment.

**Figure 1:**
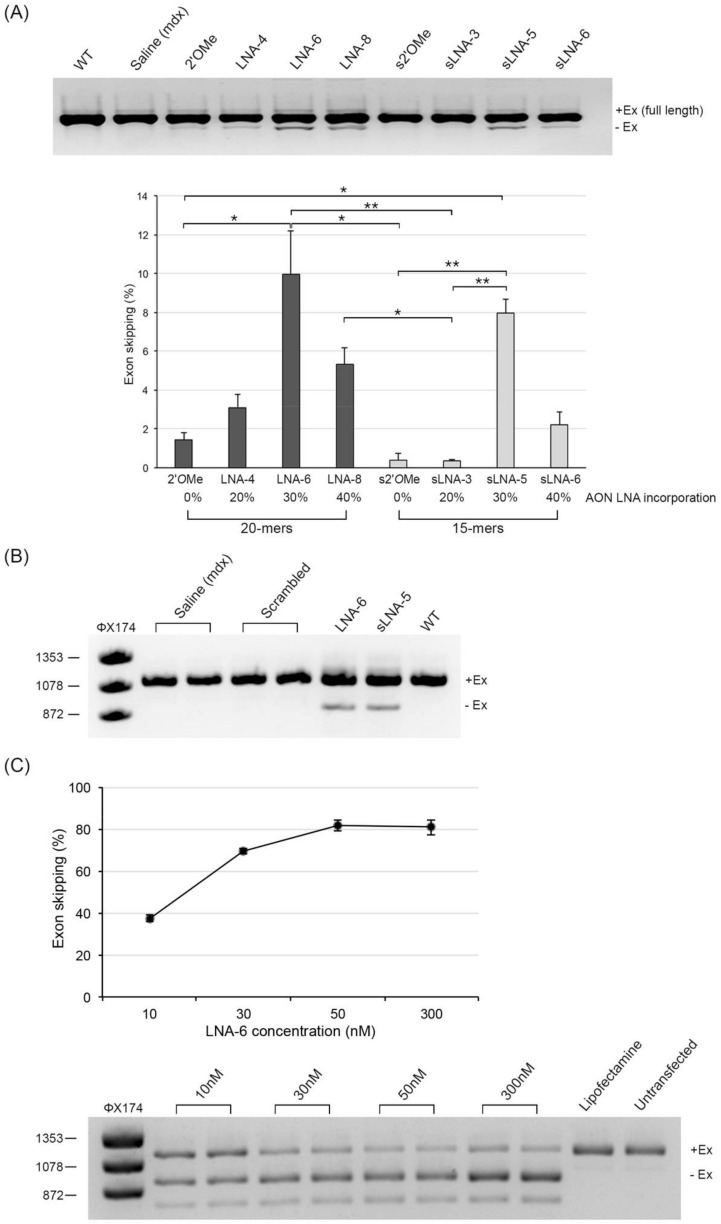
Direct comparison of exon skipping efficiencies of LNA/2′*O*Me chimeras and corresponding 2′*O*Me analogues in vivo. (**A**) RT-PCR analysis of unskipped (+Ex) and exon 23-skipped (-Ex) dystrophin mRNAs in TA muscles of mdx mice treated with an equimolar dose (4.35 nmol) of the indicated AON or saline only (representative image shown). Dystrophin expression in a WT control is also shown. Quantification of exon 23-skipped mRNA is expressed as the percentage of total dystrophin mRNA. n = 3–4 mice per group; values are mean ± SD. Square brackets and asterisks indicate statistically significant differences using Dunn’s multiple comparisons test followed by Benjamini–Hochberg adjustment. * denotes *p* ≤ 0.05 and ** denotes *p* ≤ 0.01. (**B**) Detection of exon 23-skipped dystrophin mRNA in TA muscles of mdx mice injected with an equimolar dose of a scrambled LNA/2′*O*Me sequence (n = 2). The contralateral TA was injected with saline only. Positive exon skipping control AONs (LNA-6 and sLNA-5) and a WT control mouse are also shown. (**C**) Percentage exon 23 skipping in isolated primary mdx myoblasts treated with increasing concentrations of 20mer LNA-6 AON (30% LNAs). RNA extraction was carried out 24 h post transfection. Values are mean ± SD from three independent transfection experiments.

Kruskal–Wallis analysis rendered an overall *p*-value of 0.00028, demonstrating statistically significant difference(s) among the efficiencies of the various AONs. Overall, 20mer 2′OMe and 20mer LNA/2′OMe AONs (LNA-4, LNA-6 and LNA-8) induced higher levels of exon 23 skipping compared to their respective 15mer analogues (s2′OMe, sLNA-3, sLNA-5 and sLNA-6; Figure 1A). AONs LNA-6 (20mer) and sLNA-5 (15mer), exhibiting an LNA incorporation of 30%, yielded the highest levels of exon 23 skipped RNA (10 and 8% of total dystrophin RNA, respectively), followed by 20mer AON LNA-8 consisting of 40% LNAs (5.3% exon skipping). The efficiencies of LNA-6 and sLNA-5 were found to be statistically significantly different when compared to 2′OMe, s2′OMe or sLNA-3 separately, using Dunn’s pairwise test for multiple comparisons. While the truncation of LNA-6 (30% LNA) to its shorter analogue sLNA-5 resulted in a relatively small decrease in exon skipping activity (10 versus 8%, respectively), truncation of LNA-8 (40% LNA) to generate AON sLNA-6 considerably reduced the percentage of exon skipped RNA by more than half (5.3 versus 2.2%, respectively). Taken together, these findings demonstrate that a relatively high incorporation of LNA nucleotides (≥40%) in a short antisense construct significantly reduces AON potency. Nonetheless, a 20% inclusion of LNA nucleotides to the 2′OMe and s2′OMe sequences showed minimal to no improvement in AON activity, respectively, demonstrating that an LNA incorporation of 30%, evenly distributed at every third or fourth position, is the optimal design to achieve maximal exon skipping efficiency.

To confirm on-target specificity, a scrambled LNA/2′OMe sequence with similar percentage nucleotide composition and 33% LNA inclusion was also injected into the TA muscle of mdx mice under the same experimental conditions. In contrast to our LNA-6 and sLNA-5 AONs, no skipping of mouse dystrophin exon 23 was observed following treatment with the scrambled sequence (Figure 1B). Furthermore, to determine the half maximal effective concentration (EC50) of our most potent AON, isolated primary mdx myoblasts were treated with increasing concentrations of LNA-6 (10, 30, 50 and 300 nM) and evaluated for exon 23 skipping 24 h post transfection. LNA-6 induced skipping of exon 23 in a dose-dependent manner, reaching highest efficiency (82%) at 50 nM. The EC50 value for LNA-6 was calculated at 13.9 nM (Figure 1C).

### 2.2. Evaluation of Dystrophin Restoration after Intramuscular Delivery of Chimeric LNA/2′OMe AONs in Mdx Mice

#### 2.2.1. Immunofluorescence: Number of Dystrophin Positive Fibres in Muscle Section

**Figure 2:**
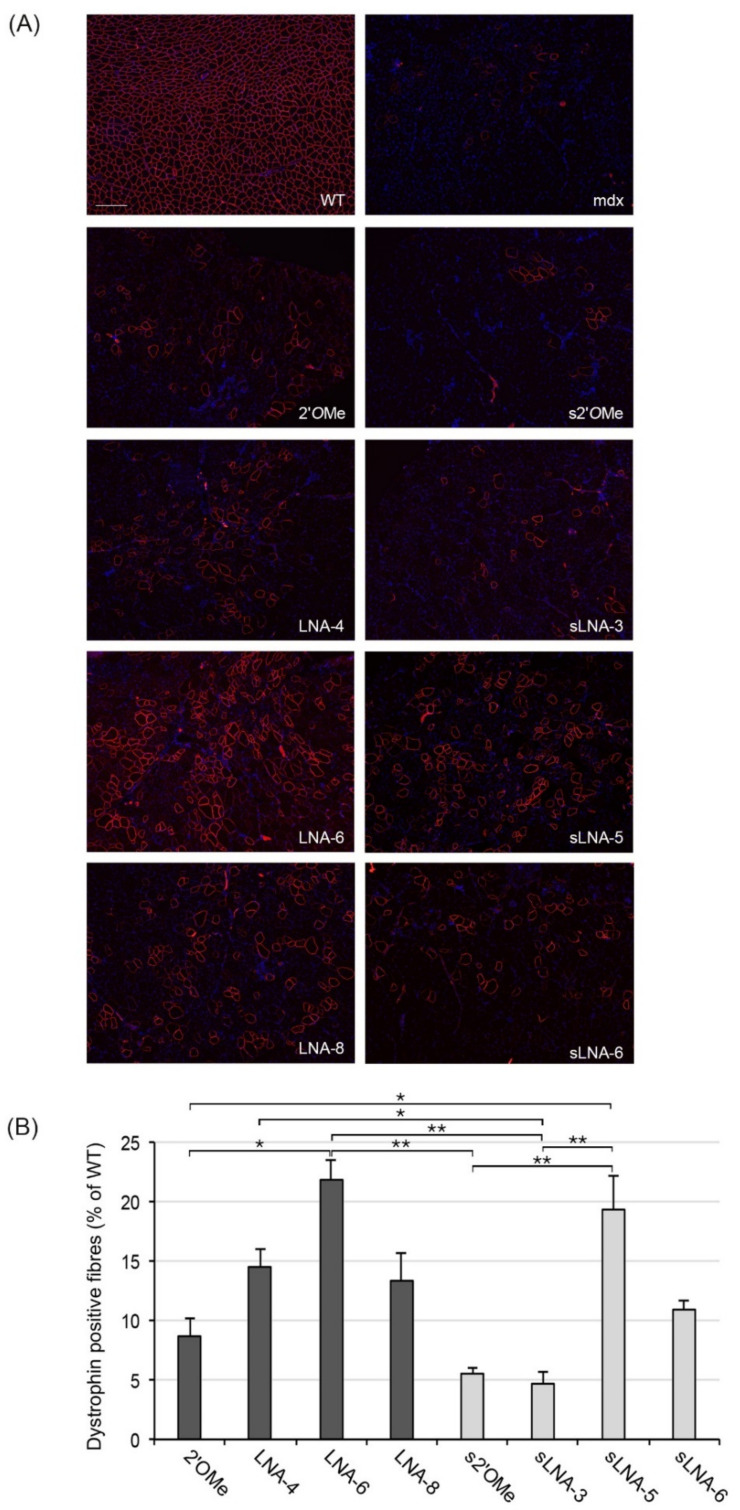
Evaluation of dystrophin restoration by immunofluorescence. (**A**) Representative images of dystrophin immunostaining on transverse muscle cryosections from a WT control and mdx mice treated with an intramuscular injection of the indicated AONs or saline only (mdx; scale bar = 100 µm). (**B**) Quantification of the number of dystrophin positive fibres, expressed as a percentage of the WT, in TA muscle cross sections from mdx mice treated with an intramuscular injection of the indicated AONs. n = 3 mice per group; values are mean ± SD. Square brackets and asterisks indicate statistically significant differences using Dunn’s multiple comparisons test followed by Benjamini–Hochberg adjustment. * denotes *p* ≤ 0.05 and ** denotes *p* ≤ 0.01.

We subsequently evaluated the efficiency of LNA/2′*O*Me AONs to induce dystrophin restoration in vivo, by immunofluorescence staining of TA cryosections using a polyclonal antibody against the C-terminal domain of dystrophin. Dystrophin restoration was measured as the number of dystrophin-positive fibres in the whole muscle section and reported as a percentage of the WT (Figure 2). Kruskal–Wallis analysis rendered an overall *p*-value of 0.0001, demonstrating statistically significant difference(s) among the efficiencies of the various AONs. In agreement with the exon skipping data, 20mer LNA-6 and 15mer sLNA-5 AONs, both consisting of 30% LNAs, showed the highest percentages of dystrophin positive fibres (22 and 19% of WT, respectively), while 15mer AONs s2′*O*Me and sLNA-3 (20% LNAs) induced significantly lower dystrophin restoration corresponding to ~5% of the WT (adjusted *p*-values ≤ 0.01 for each pairwise comparison: LNA-6–s2′*O*Me, LNA-6–sLNA-3, sLNA-5–s2′*O*Me and sLNA-5–sLNA-3). LNA-8 and sLNA-6, exhibiting the highest percentage of LNA incorporation, were less efficient compared to their respective AONs containing 30% LNAs, underscoring again the negative impact of an increased LNA inclusion on AON activity. Notably, while inducing minimal (2%) skipping of exon 23 in TA muscles, AON LNA-4 containing 20% LNAs showed a 14% increase in dystrophin positive fibres relative to the WT. This result reflects on the interpretive bias of the immunofluorescence analysis since the number of dystrophin positive fibres is not necessarily proportional to the levels of dystrophin expression; i.e., a larger number of dystrophin positive fibres may respectively express lower levels of the protein and, conversely, a smaller number of dystrophin positive fibres may respectively express higher levels of the protein.

#### 2.2.2. Mass Spectrometry Quantification of Dystrophin

**Figure 3:**
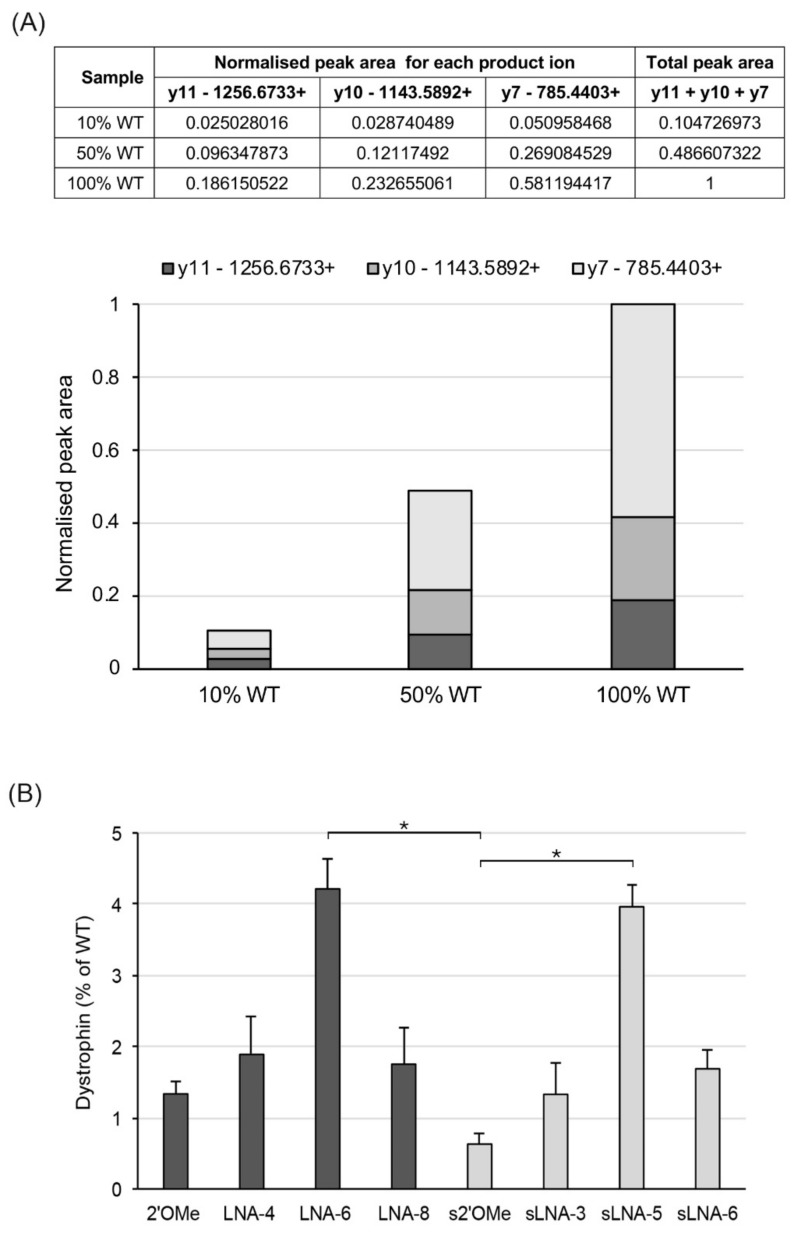
Targeted MS quantification of total dystrophin protein in WT and AON-treated mdx TA muscles. (**A**) Quantification of the IFLTEQPLEGLEK peptide in WT protein concentration standards (10, 50 and 100%) showing the peak area values of its three product ions (y11+, y10+ and y7+). The peak area of each product ion, as well as the sum peak area of all three transitions (last column), increase proportionally to the concentration of the WT standard (table values and bar chart). (**B**) Quantification of dystrophin expression, plotted as a percentage of the WT, in TA muscles of mdx mice after intramuscular delivery with the indicated AONs. n = 3 mice per group; values are mean ± SD. Square brackets and asterisks indicate pairwise *p*-values ≤ 0.05 using Dunn’s multiple comparisons test followed by Benjamini–Hochberg adjustment.

To confirm dystrophin expression, we carried out a targeted mass spectrometry (MS)-based protein quantification assay using aliquots of 100 ng total protein extract from WT control and AON-treated mdx mice. Protein quantification was carried out using two custom dystrophin peptides: IFLTEQPLEGLEK, used as quantifier, and LLAEELPLR, used as qualifier. To assess linearity of our assay, three WT concentration standards were prepared (10, 50 and 100% of WT extract) and used for the relative quantification of dystrophin levels in AON-treated mdx samples. As evident in Figure 3A, our assay shows linearity over a wide dynamic range (10 to 100%): peak area values for each product ion of the quantifier peptide increase proportionally to the input amount of WT protein, i.e., a 5-fold increase in peak area is observed from 10 to 50% of input protein and a 2-fold increase from 50 to 100%. Therefore, dystrophin levels in AON-treated mdx samples were determined using the sum peak area of all three product ions of the quantifier peptide.

As expected, AONs LNA-6 and sLNA-5 consisting of 30% LNAs induced the highest levels of dystrophin restoration (4.2 and 4% of WT, respectively; Figure 3B, Figure S1), followed by AONs LNA-8 and sLNA-6 with 40% LNA inclusion and 20mer LNA-4 containing 20% LNAs; all of the latter three showed similar levels of restored dystrophin expression corresponding to ~1.8% of the WT (overall *p*-value of 0.009 using Kruskal–Wallis analysis). Notably, efficiency differences among LNA/2′*O*Me chimeras were more pronounced for exon 23 skipping (Figure 1A) compared to rescued dystrophin expression: at the RNA level, increasing the percentage of LNA composition from 20 to 30% resulted in a 5- and 16-fold increase in AON activity for the 20mer and 15mer AONs, respectively, compared to a 2- and 3-fold rise when evaluating protein restoration by MS. Similarly, while 20mer and 15mer LNA/2′*O*Me AONs with 20 and 40% LNA compositions showed similar levels of rescued protein expression, exon skipping activity of LNA-8 (20mer with 40% LNAs) and sLNA-6 (15mer with 40% LNAs) increased by 2.5- and 5-fold, respectively, compared to their corresponding AONs with 20% LNA composition. These findings reflect highly on the sensitivity of the techniques and how RNA data may correlate with corresponding data at the protein level.

#### 2.2.3. Western Blot Quantification of Dystrophin

**Figure 4:**
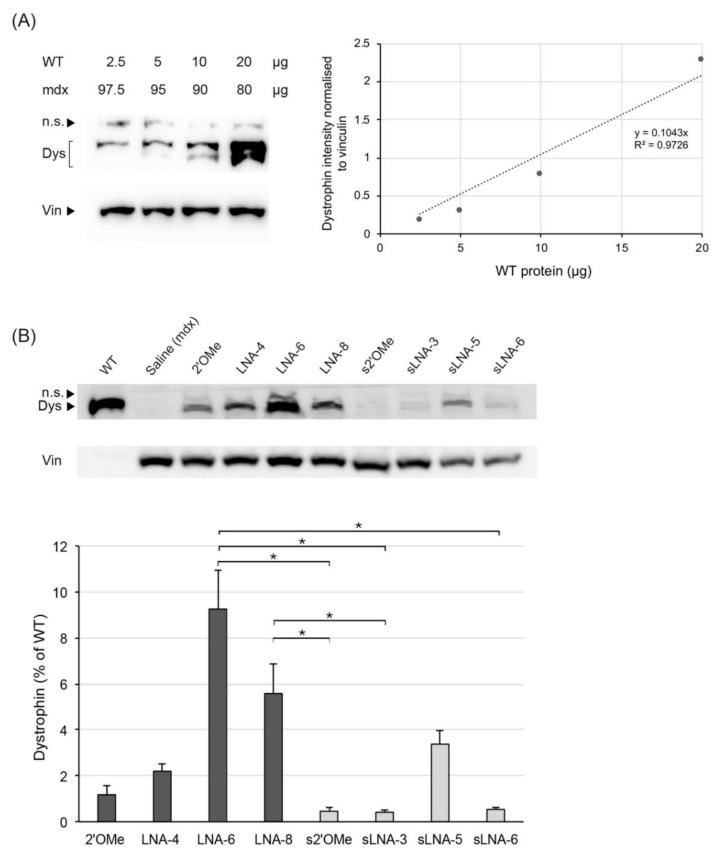
WB quantification of total dystrophin protein in WT and AON-treated mdx TA muscles. (**A**) A four-point standard curve for WT dystrophin was created, with concentration range of 2.5 to 20%, by spiking pooled WT protein extracts into pooled mdx lysates to maintain equal loading of protein (100 μg). The dystrophin signal (bottom bands in top gel; n.s. = non-specific band) normalized to the intensity of the vinculin signal (bottom gel) was graphed in linear format. The identified equation and R2 value of the linear regression analysis are shown on the graph. (**B**) Detection and quantification of total dystrophin protein in TA muscles of mdx mice, 2 weeks after intramuscular injection with the indicated AONs (representative image shown). To minimize saturation issues on the gel, the dystrophin band signal in the WT lane corresponds to only 10 μg of loaded protein (i.e., 10% of total protein extract loaded for saline and AON-treated samples). Dystrophin band signals were normalised to the intensity of the respective vinculin signals and the amount of rescued protein, expressed as a percentage of the WT, was calculated using the equation identified in the linear regression analysis. n = 3 mice per group; values are mean ± SD. Square brackets and asterisks indicate pairwise *p*-values ≤ 0.05 using Dunn’s multiple comparisons test followed by Benjamini–Hochberg adjustment.

Dystrophin restoration was further evaluated by chemiluminescent Western blotting using a multi-point standard curve to assess for potential oversaturation on the gels, which would lead to overestimation of protein levels at the low end of the range. A four-point standard curve was constructed by spiking quantified aliquots of a WT protein extract (2.5, 5, 10 and 20 μg) into mdx lysates to produce 2.5, 5, 10, and 20% concentration standards, respectively (Figure 4A). Data were analysed by linear regression, generating a coefficient of determination (R2) value of 0.9726, and the identified equation (y = 0.1043x) was used to calculate the amount of restored dystrophin in AON-treated mdx samples. The standard curve demonstrates good linearity of the chemiluminescent signal over the indicated concentration range (2.5 to 20%), which corresponds to the percentage range of dystrophin positive fibres observed in muscles sections of AON-treated mice (Figure 3B). In contrast to the protein expression profiles obtained by immunofluorescence and targeted MS analysis, chemiluminescent detection of Western blot products revealed a 2.8- and 11-fold decrease in the efficiencies of 15mer LNA/2′*O*Me AONs consisting of 30 or 40% LNAs, respectively, compared to their corresponding 20mer analogues (Figure 4B; overall *p*-value of 0.0026 using Kruskal–Wallis analysis). Nonetheless, in agreement with the exon skipping data, 20mer and 15mer LNA/2′*O*Me AONs consisting of 30% LNAs were more potent than their respective 2′*O*Me/PS and LNA/2′*O*Me counterparts exhibiting a higher (40%) or lower (20%) percentage LNA composition.

## 3. Discussion

AON-mediated exon skipping has been in the forefront of DMD therapeutic research for the past 20 years. Two chemically modified oligonucleotide designs, a PMO and a 2′*O*Me/PS AON, have entered clinical trials, with the former receiving provisional FDA approval based on minor increases in rescued dystrophin expression. By contrast, the 2′*O*Me/PS AON was associated with adverse systemic and injection-site reactions and was therefore withdrawn from consideration. Attempting to limit the toxicity associated with the PS modification, Le et al. designed a series of truncated 2′*O*Me/PS AONs, relying on the introduction of LNA nucleotide monomers to retain a high RNA binding affinity. Indeed, the introduction of four LNAs in a 14mer 2′*O*Me/PS AON, showing no exon skipping activity, significantly improved the yield of exon 23-skipped RNA in H2K mdx mouse myotubes [44]. To further evaluate the optimal length and percentage chemistry composition of LNA/2′*O*Me chimeras for DMD therapy, we created a series of 15mer and 20mer AONs, with variable LNA compositions (20–40%), and compared their efficiencies in inducing exon 23 skipping and rescued protein expression in locally injected muscles of mdx mice.

When optimising novel oligonucleotide designs for clinical therapy, the sensitivity of the various methodologies is crucial for determining subtle differences in the efficiency of the tested AONs. In the present study, AON efficacy was assessed at RNA level by densitometric analysis of PCR products and at protein level by semi-quantitative immunofluorescent analysis, quantitative MS analysis and quantitative chemiluminescent WB. While comparing the data obtained by the various methodologies, we observed that efficiency differences among LNA/2′*O*Me chimeras with variable LNA compositions were significantly more prominent for exon skipping percentages analysed by RT-PCR and rescued protein levels determined by WB. Considering that dystrophin gene expression is rather low in healthy tissues, accurate quantification of low exon-skipping samples would be particularly challenging. The highest exon skipping percentage reported in this study was 10%, for 20mer LNA-6 (30% LNAs), and the lowest was 0.4%, for 15mer AONs s2′*O*Me and sLNA-3 (20% LNAs). In a comparative study of different methodologies to quantify exon skipping percentages in AON-treated mdx muscle tissues, samples with exon skipping percentages between 4 and 10%, as determined by an absolute quantification method, showed a 30–50% value variation following densitometric analysis of ethidium bromide-stained PCR products [48]. Notably, this was not observed for samples with reference exon skipping values between 28 and 30%. Therefore, while relatively accurate, densitometric analysis of PCR products could lead to an under- or over-estimation of the real values in samples with low exon skipping percentages.

While LNA-6 (20mer with 30% LNAs) and sLNA-5 (15mer with 30% LNAs) AONs showed similar levels of rescued dystrophin expression by MS analysis, WB quantification of dystrophin levels revealed a 2.8-fold decrease in the efficiency of the sLNA-5 AON compared to its longer analogue. WB followed by chemiluminescent detection has served as a golden standard for dystrophin quantification in both clinical and pre-clinical studies; yet, the robustness of this method is limited by poor transfer qualities, saturation issues and poor reproducibility. During an assessment of dystrophin levels in patients treated with eteplirsen, researchers identified an average coefficient of variation of 16% while performing sample duplicates in separate gels [49]. This highlights the importance of performing at least two technical replicates per biological replicate, something that was not carried out in the present study. Furthermore, while MS shows higher reproducibility compared to WB, it has been reported to demonstrate low resolution for dystrophin values under 5% of WT (dystrophin values obtained by MS analysis in the present study were between 0.5 and 4.5%) [49]. Considering all of the aforementioned limitations, and based on the results of the present study, we recommend that dystrophin restoration should always be quantified using all three methodologies, to allow for a more robust correlation between RNA and protein expression profiles and thus a more accurate comparison of AON efficiencies during pre-clinical assessment.

Collectively, the data obtained demonstrate that 15mer and 20mer LNA/2′*O*Me AONs consisting of 30% LNAs, evenly distributed at every third or fourth position, are significantly more potent in inducing exon 23 skipping and restoring dystrophin expression in locally injected mdx muscles, compared to a previously tested 2′*O*Me AON and LNA/2′*O*Me chimeras with lower or higher LNA compositions. In agreement with current findings, recently published work from our laboratory has shown that 14–18mer LNA/2′*O*Me AONs with a 33% LNA composition and a full PS backbone, targeting the expanded CTG-repeat mutation that causes DM1, were more efficient in correcting DM1 molecular defects in vitro compared to an 18mer LNA/2′*O*Me AON containing half the number of LNA nucleotides and LNA/DNA mixmers of similar or higher percentage LNA composition [45]. More importantly, systemic delivery of the 14mer and 18mer LNA/2′*O*Me chimeras in DM1 mice resulted in relatively high levels of AON accumulation in skeletal muscles, diaphragm and heart tissue, with no profound histological toxicity.

Efficient delivery of AONs to the cardiac tissue is of great therapeutic relevance, as almost all DMD patients will manifest signs of cardiomyopathy by the age of 18. Consequently, failure of dystrophin restoration in cardiac tissue, in the presence of rescued protein expression in skeletal muscles, may exacerbate any subclinical heart failure due to increased voluntary muscle activity [50,51]. Delivery to the heart and skeletal muscle tissues has been improved by conjugating positively charged arginine-rich peptides to charge neutral PMOs [52,53,54]. Therefore, future systemic evaluation of our LNA/2′*O*Me chimeras may be further exploited using a combination of muscle-homing, hydrophobic ligands that would potentially enhance AON accumulation and efficacy in all target tissues. While intramuscular delivery may be sufficient to screen a larger number of AON designs with different chemical compositions, it has limited capacity in providing solid evidence on the therapeutic efficacy of an antisense sequence. Systemic delivery of our two most potent AONs, LNA-6 and sLNA-5, is further warranted to evaluate the benefit-toxicity profile of the respective LNA/2′*O*Me sequences for DMD therapy.

## 4. Materials and Methods

### 4.1. Antisense Oligonucleotides

All AONs used in the study, listed in Table 1, were designed and produced by the laboratory of Professor Jesper Wengel. We have chosen the most efficient 2′*O*Me/PS AON sequence from previous studies, which targets the donor splice site of exon 23 in the mouse dystrophin pre-mRNA [46,47]. For simplicity of nomenclature, the AON was named 2′*O*Me. A varying number of LNA nucleotides was introduced throughout the 20mer 2′*O*Me sequence to generate chimeric LNA/2′*O*Me AONs with LNA percentage incorporations of 20, 30 and 40%. These were named LNA-4, LNA-6 and LNA-8, respectively, to refer to the number of LNA nucleotides introduced in the sequence. Truncated, 15mer analogues of the 2′*O*Me and LNA/2′*O*Me oligonucleotides were also generated, distinguished by the letter ‘s’ at the beginning of the AON name. All AONs were kept on a fully modified PS backbone. An 18mer CAG-repeat LNA/2′*O*Me AON with a full PS backbone and an LNA incorporation of 33% was also included in the study to serve as a control scrambled sequence.

### 4.2. Animal Experiments

Dmd mdx mutant mice (also known as mdx), carrying a spontaneous nonsense mutation (C to T transition) in exon 23 of the mouse dystrophin gene, where purchased from Jackson Laboratories. Animal studies were carried out at the Transgenic Mouse Facility of the Cyprus Institute of Neurology & Genetics, following evaluation by the “National Committee for the Protection of Animals Used for Scientific Purposes” and approval by the Cyprus Veterinary Services (project license approval number: CY/EXP/PR.L2/2014). The AONs were delivered into the TA muscle of 8-week-old mdx mice, under general anaesthesia. For the injections, 4.35 nmoles of each AON, calculated based on the molecular weight, were diluted in 30 μL total volume of saline. The contralateral TA was injected with 30 μL of saline only. Two weeks after the injection, the mice were sacrificed by cervical dislocation, the muscles were isolated and snap-frozen in liquid nitrogen-cooled isopentane, and stored at −80 °C for further experimentation. Aged-matched C57BL10 mice (wild type, WT) that lack the DMD mutation were used as a control.

### 4.3. RNA Extraction and RT-PCR

Frozen tissues were cryosectioned in the transverse plane at 5 μm thickness and 100 μm intervals. Intervening muscle sections were collected for immunofluorescence, Western blot and targeted mass spectrometry analysis. Sections were homogenized in TRIzol reagent using a Precellys tissue homogenizer and total RNA was then extracted according to the manufacturer’s instructions. RT-PCR was carried out using 300 ng of total RNA in a 50 μL reaction, using the One-step RT-PCR kit (Qiagen, Hilden, Germany) and external primers Ex20F (5′-CAGAATTCTGCCAATTGCTGAG-3′) and Ex26R (5′-TTCTTCAGC TTGTGTCATCC-3′). The cycling conditions were 95 °C for 1 min, 55 °C for 1 min and 72 °C for 2 min for 30 cycles. PCR products were examined by electrophoresis on a 2% agarose gel and densitometric analysis was carried out using Image J. The percentage of exon skipping was calculated as the percentage of transcripts in which exon 23 is skipped relative to the sum of non-skipped and exon 23-skipped dystrophin transcripts. The RT-PCR experimental procedure was implemented according to Spitali et al., 2010: “Accurate quantification of dystrophin mRNA and exon skipping levels in Duchenne muscular dystrophy” [48]. To detect exon skipping in AON-transfected primary mdx myoblasts (see section below), total RNA was extracted by TRIzol reagent and 300 ng were subjected to RT-PCR analysis using 27 cycles of amplification instead of 30.

### 4.4. Isolation of Primary Mdx Myoblasts and Transfection

The protocol for isolating satellite cells from single muscle fibres was adapted from Rosenblatt and colleagues [55]. In brief, extensor digitorum longus (EDL) muscles were isolated from 8-week-old mdx mice and incubated in 0.2% type I collagenase (Sigma-Aldrich, St. Louis, MS, USA) in Dulbecco’s Modified Eagle Medium (DMEM, high glucose, pyruvate, no glutamine; Thermo Fisher Scientific, Waltham, MA, USA), supplemented with 1× penicillin-streptomycin (PS; Thermo Fisher Scientific) and 1× glutamax solution (Thermo Fisher Scientific), at 37 °C for 1 h. Following digestion, single muscle fibres were liberated by repeatedly triturating the EDL muscle using a wide-mouth glass pipette. Viable single muscle fibres were carefully transferred to a new culture dish, in three rounds of washing, in order to discard hypercontracted fibres and tissue debris. Finally, viable muscle fibres were transferred to a new culture dish coated with 1 mg/mL Matrigel (Corning) in DMEM (1× PS/1× glutamax) and incubated in ‘plating medium’ (DMEM supplemented with 1× PS, 1× glutamax, 10% horse serum (HS; Thermo Fisher Scientific), 0.5% chick embryo extract (CEE; MP Biomedicals)) at 37 °C for 3 days, to allow myogenic satellite cells to dissociate from their fibre. Three days after plating, the muscle fibres were removed from the dish and the ‘plating medium’ was replaced with ‘proliferation medium’ consisting of 20% fetal bovine serum (FBS; Thermo Fisher Scientific), 10% HS, 1% CEE and 2.5 ng/mL recombinant murine fibroblast growth factor-basic (FGF-b; PeproTech, London, UK) in DMEM (1× PS/1× glutamax). Myogenic cells were allowed to grow in 5% CO^2^ and 37 °C for 2–3 days, before plating for transfection. For the dose–response study, primary mdx myoblasts were seeded in 12-well plates, at 60% confluency, and transfected with increasing concentrations of AON LNA-6 (10, 30, 50 and 300 nM), complexed with Lipofectamine 3000 reagent in Opti-MEM Reduced Serum Medium. Exon skipping efficiency was measured 24 h post transfection by RT-PCR for exon 23 skipping. Gel bands were measured using Image J and half-maximal effective concentration (EC50) was calculated using the sigmoid Emax dose–response model in the DoseFinding R package (Date: 5 July 2019).

### 4.5. Immunofluorescence

Transverse cryosections of 7 µm thickness were examined for dystrophin expression using a rabbit polyclonal anti-dystrophin antibody (1:400 dilution, RB-9024-P Thermo Scientific), which was detected using the Alexa Fluor 594 goat anti-rabbit antibody (molecular probes ref 37117). Dystrophin-labelled sections were imaged at low magnification (5×) and composite images of the entire transverse section were constructed from overlapping images. The number of dystrophin positive fibres in the entire muscle cross section was then counted using the cell count function in Image J (3 sections per TA muscle were counted). The percentage of dystrophin positive fibres for each AON was calculated relative to the average number of dystrophin positive fibres in WT control sections.

### 4.6. Sample Preparation for Mass Spectrometry

Intervening cryosections from WT control and AON-treated mdx muscle tissues were lysed in protein homogenization buffer (refer to Section 4.8) and aliquots of total protein extract were further processed using a modified filtered-aided sample preparation (FASP) protocol [56]. Briefly, 100 ng of protein was transferred to a centrifugal filter (Pall Nanosep, 30 kDa MWCO), washed with urea buffer, then reduced with 100 µL DTT (8 mM in 50 mM ammonium bicarbonate) for 15 min at 60 °C and alkylated with 100 µL iodoacetamide (50 mM in 50 mM ammonium bicarbonate) for 20 min in the dark, at room temperature. Finally, the samples were washed with 100 µL of 50 mM ammonium bicarbonate (×3) and digested with 2 µg trypsin (proteomics grade, Roche Diagnostics GmbH, Mannheim, Germany) at 37 °C for 18 h. Peptides were collected by centrifugation for 10 min at 11,000× *g*, acidified with TFA to a final concentration of 0.2% and desalted using reverse phase solid phase extraction cartridges (Sep-Pak C18, Waters, UK). Eluates were lyophilized using a centrifugal vacuum concentrator. Peptide pellets were re-dissolved in 1% acetonitrile, 0.1% formic acid to yield an approximate concentration of 250 ng/µL (determined by NanoDrop measurement at 280 nm).

### 4.7. Mass Spectrometry Analysis

Custom dystrophin peptides LLAEELPLR and IFLTEQPLEGLEK were purchased from GenScript Biotech (The Netherlands). Peptides were suspended in 1% LC-MS grade acetonitrile containing 0.1% formic acid, pooled together and further diluted to a final concentration of 10 fmol/µL. To determine the retention time of each peptide, 5 µL of the peptide mix were loaded onto a C18 column (Acquity UPLC M-Class Peptide CSH, 75 µm × 250 mm, 1.7 µm) and separated at a flow rate of 300 nL/min using a nanoAcquity UPLC system (Waters, UK). The mobile phases consisted of 0.1% formic acid in water (mobile phase A) and 0.1% in acetonitrile (mobile phase B). Peptides eluted with a linear gradient from 5% B to 31% B over 57 min (elution profile is shown in Supplementary Table S1). An HD-MRM (High-Definition Multiple Reaction Monitoring) MS assay, which is based on ion mobility mass spectrometry technique, was developed. The method employs additional separation of peptide fragments based on their collisional cross section (CCS), followed by high resolution MS detection and is implemented on an ion mobility-enabled quadrupole time-of-flight hybrid mass spectrometer [57]. The targeted MS assay was implemented on a Synapt G2Si HDMS instrument equipped with the NanoLockSpray source and operated on the HD-MRM acquisition mode. Fragmentation was performed on the Trap T-Wave collision cell and fragment ions were monitored using the Wideband Enhancement mode. For each peptide, the three highest intensity transitions were monitored. In particular, the transition 758.9165 > 785.4403, 758.9165 > 1143.5892 and 758.9165 > 1256.6733 as well as the 527.3188 > 827.4621, 527.3188 > 756.4250 and 527.3188 > 940.5462, were used to monitor the IFLTEQPLEGLEK and the LLAEELPLR peptides, respectively. Optimized instrument parameters are shown in the Supplementary Table S2. Data analysis and interpretation were performed using Skyline [58].

### 4.8. Western Blot

Intervening cryosections from untreated and AON-treated mdx TA muscles were collected in Precellys CK14 tubes and lysed in 120 µL of homogenization buffer containing 1.25 mM Tris-HCl pH 6.8, 10% Glycerol, 4% SDS, 4 M Urea, 10% b-mercaptoethanol and 200 µL protease inhibitor (25x). Protein extracts were then denatured at 95 °C for 5 min and centrifuged to collect the supernatant. A sample of the supernatant mixture was acetone-treated and used to determine protein concentration using the Bradford assay (Biorad). In total, 100 µg of protein extract from treated and untreated TA muscles were loaded on 6% SDS-PAGE gels. The samples were electrophoresed for 3.5 h at 80 V and blotted to a PVDF membrane at 30 V overnight at 4 °C. The membrane was probed overnight at 4 °C with 1:500 dilution of rabbit anti-dystrophin antibody (Thermo fisher 9024-P1) in 5% skimmed milk. The bound antibody was detected by incubation with a horseradish peroxidise conjugated goat anti-rabbit antibody (1:5000 dilution, Jackson laboratories) in 5% milk in PBS-T and the Lumi Sensor chemiluminescent HRP Substrate kit (L00221V300). The membranes were also probed overnight at 4 °C with rabbit anti-vinculin (1:1000 dilution, Abcam ab73412) in 5% skimmed milk, which served as a loading control. Band intensities were analysed using Image J and dystrophin values were normalised with respect to vinculin.

To construct a standard curve for WT dystrophin, aliquots of a pooled (4 TA muscles) WT protein extract (2.5, 5, 10 and 20 μg) were spiked into aliquots of a pooled mdx extract (97.5, 95, 90 and 80 μg, respectively) to maintain an equal loading of protein (100 μg). Protein homogenization, sample preparation and Western blotting were carried out as described above. Dystrophin band signals were normalized to the intensity of the respective vinculin signal and data were analysed by linear regression. The identified equation of the linear regression line was used to calculate the amount of restored dystrophin in AON-treated mdx samples.

### 4.9. Statistical Analysis

All data are expressed as mean ± standard deviation (SD). Statistical significance was determined using Kruskal–Wallis one-way analysis of variance followed by Dunn’s (1964) pairwise test for multiple comparisons. *p*-values were adjusted using the Benjamini–Hochberg (false discovery rate) method.

## 5. Conclusions

In vivo screening of different LNA/2′*O*Me antisense designs for targeted exon 23 skipping in mdx TA muscles revealed that AONs consisting of 30% LNAs are more potent in inducing exon 23 skipping and restoring dystrophin protein expression, compared to a 2′*O*Me-modified AON and LNA/2′*O*Me chimeras with lower (20%) or higher (40%) LNA compositions. Our data further demonstrate the importance of using parallel techniques (i.e., immunohistochemistry, mass spectrometry and Western blotting) for the quantification of dystrophin protein restoration, to allow for a more robust correlation between RNA and protein expression profiles and a more accurate comparison of AON efficiencies during pre-clinical assessment. Systemic evaluation is now warranted to prove the therapeutic potential of our most potent LNA/2′*O*Me design, comprising of 30% LNAs, evenly distributed at every third of fourth position of the AON sequence.

## Figures and Tables

**Table 1:** Oligonucleotide nomenclature, sequence and chemistries.

AON ID	Sequence (5′ → 3′) and Modifications	Length	% LNA
2′*O*Me	mG* mG* mC* mC* mA* mA* mA* mC* mC* mU* mC* mG* mG* mC* mU* mU* mA* mC* mC* mU	20mer	0
LNA-4	mG* G* mC* mC* mA* mA* A* mC* mC* mU* mC* G* mG* mC* mU* mU* A* mC* mC* mU	20mer	20
LNA-6	mG* G* mC* mC* A* mA* mA* C* mC* mU* mC* G* mG* mC* mU* T* mA* mC* C* mU	20mer	30
LNA-8	mG* G* mC* mC* A* mA* A* mC* C* mU* mC* G* mG* C* mU* T* mA* mC* C* mU	20mer	40
s2′*O*Me	mA* mA* mC* mC* mU* mC* mG* mG* mC* mU* mU* mA* mC* mC* mU	15mer	0
sLNA-3	mA* mA* mC* C* mU* mC* mG* G* mC* mU* mU* A* mC* mC* mU	15mer	20
sLNA-5	mA* A* mC* mC* T* mC* mG* G* mC* mU* T* mA* mC* C* mU	15mer	33
sLNA-6	A* mA* mC* C* mU* C* mG* mG* C* mU* T* mA* mC* C* mU	15mer	40
CAG-scrambled	C* mA* mG* C* mA* mG* C* mA* mG* C* mA* mG* C* mA* mG* C* mA* mG	18mer	33
**bold** = LNA, m = 2′*O*Me, * = PS linkages			

## Supplementary Materials

The following are available online at https://www.mdpi.com/article/10.3390/ph14111113/s1, Figure S1: Representative HD-MRM chromatograms of selected transitions, Table S1: UPLC Elution profile and LC parameters, Table S2: HD-MRM transitions and MS parameters for dystrophin peptides.

## References

[1] Muntoni F., Torelli S., Ferlini A. (2003). Dystrophin and mutations: One gene, several proteins, multiple phenotypes. Lancet. Neurol..

[2] Koenig M., Hoffman E.P., Bertelson C.J., Monaco A.P., Feener C., Kunkel L.M. (1987). Complete cloning of the Duchenne muscular dystrophy (DMD) cDNA and preliminary genomic organization of the DMD gene in normal and affected individuals. Cell.

[3] Helderman-van den Enden A.T.J.M., Straathof C.S., Aartsma-Rus A., den Dunnen J.T., Verbist B.M., Bakker E., Verschuuren J.J., Ginjaar H.B. (2010). Becker muscular dystrophy patients with deletions around exon 51; a promising outlook for exon skipping therapy in Duchenne patients. Neuromuscul. Disord. NMD.

[4] Mann C.J., Honeyman K., Cheng A.J., Ly T., Lloyd F., Fletcher S., Morgan J.E., Partridge T.A., Wilton S.D. (2001). Antisense-induced exon skipping and synthesis of dystrophin in the mdx mouse. Proc. Natl. Acad. Sci. USA.

[5] Aartsma-Rus A., Janson A.A., Kaman W.E., Bremmer-Bout M., den Dunnen J.T., Baas F., van Ommen G.J., van Deutekom J.C. (2003). Therapeutic antisense-induced exon skipping in cultured muscle cells from six different DMD patients. Hum. Mol. Genet..

[6] Aartsma-Rus A., Bremmer-Bout M., Janson A.A., den Dunnen J.T., van Ommen G.J., van Deutekom J.C. (2002). Targeted exon skipping as a potential gene correction therapy for Duchenne muscular dystrophy. Neuromuscul. Disord. NMD.

[7] Aartsma-Rus A., van Ommen G.J. (2007). Antisense-mediated exon skipping: A versatile tool with therapeutic and research applications. Rna.

[8] Yokota T., Duddy W., Partridge T. (2007). Optimizing exon skipping therapies for DMD. Acta Myol..

[9] Amantana A., Iversen P.L. (2005). Pharmacokinetics and biodistribution of phosphorodiamidate morpholino antisense oligomers. Curr. Opin. Pharmacol..

[10] Alter J., Lou F., Rabinowitz A., Yin H., Rosenfeld J., Wilton S.D., Partridge T.A., Lu Q.L. (2006). Systemic delivery of morpholino oligonucleotide restores dystrophin expression bodywide and improves dystrophic pathology. Nat. Med..

[11] Fletcher S., Honeyman K., Fall A.M., Harding P.L., Johnsen R.D., Steinhaus J.P., Moulton H.M., Iversen P.L., Wilton S.D. (2007). Morpholino oligomer-mediated exon skipping averts the onset of dystrophic pathology in the mdx mouse. Mol. Ther. J. Am. Soc. Gene Ther..

[12] Mendell J.R., Goemans N., Lowes L.P., Alfano L.N., Berry K., Shao J., Kaye E.M., Mercuri E., Eteplirsen Study G., Telethon Foundation D.M.D.I.N. (2016). Longitudinal effect of eteplirsen versus historical control on ambulation in Duchenne muscular dystrophy. Ann. Neurol..

[13] Charleston J.S., Schnell F.J., Dworzak J., Donoghue C., Lewis S., Chen L., Young G.D., Milici A.J., Voss J., DeAlwis U. (2018). Eteplirsen treatment for Duchenne muscular dystrophy: Exon skipping and dystrophin production. Neurology.

[14] Stein C.A. (2016). Eteplirsen Approved for Duchenne Muscular Dystrophy: The FDA Faces a Difficult Choice. Mol. Ther. J. Am. Soc. Gene Ther..

[15] Aartsma-Rus A., Krieg A.M. (2017). FDA Approves Eteplirsen for Duchenne Muscular Dystrophy: The Next Chapter in the Eteplirsen Saga. Nucleic Acid Ther..

[16] Aartsma-Rus A., Arechavala-Gomeza V. (2018). Why dystrophin quantification is key in the eteplirsen saga. Nat. Rev. Neurol..

[17] Khan N., Eliopoulos H., Han L., Kinane T.B., Lowes L.P., Mendell J.R., Gordish-Dressman H., Henricson E.K., McDonald C.M., Eteplirsen I. (2019). Eteplirsen Treatment Attenuates Respiratory Decline in Ambulatory and Non-Ambulatory Patients with Duchenne Muscular Dystrophy. J. Neuromuscul. Dis..

[18] Alfano L.N., Charleston J.S., Connolly A.M., Cripe L., Donoghue C., Dracker R., Dworzak J., Eliopoulos H., Frank D.E., Lewis S. (2019). Long-term treatment with eteplirsen in nonambulatory patients with Duchenne muscular dystrophy. Medicine.

[19] Shirley M. (2021). Casimersen: First Approval. Drugs.

[20] Wagner K.R., Kuntz N.L., Koenig E., East L., Upadhyay S., Han B., Shieh P.B. (2021). Safety, tolerability, and pharmacokinetics of casimersen in patients with Duchenne muscular dystrophy amenable to exon 45 skipping: A randomized, double-blind, placebo-controlled, dose-titration trial. Muscle Nerve.

[21] Muntoni F., Frank D., Sardone V., Morgan J., Schnell F., Charleston J., Desjardins C., Phadke R., Sewry C., Popplewell L. (2018). Golodirsen Induces Exon Skipping Leading to Sarcolemmal Dystrophin Expression in Duchenne Muscular Dystrophy Patients With Mutations Amenable to Exon 53 Skipping (S22.001). Neurology.

[22] ClinicalTrials.gov Identifier: NCT02310906, Phase I/II study of SRP- 4053 in DMD Patients. NCT02310906.

[23] ClinicalTrials.gov Identifier: NCT02500381, Study of SRP-4045 and SRP- 4053 in DMD Patients (ESSENCE). NCT02500381.

[24] Komaki H., Nagata T., Saito T., Masuda S., Takeshita E., Sasaki M., Tachimori H., Nakamura H., Aoki Y., Takeda S. (2018). Systemic administration of the antisense oligonucleotide NS-065/NCNP-01 for skipping of exon 53 in patients with Duchenne muscular dystrophy. Sci. Transl. Med..

[25] Dhillon S. (2020). Viltolarsen: First Approval. Drugs.

[26] Goemans N.M., Tulinius M., van den Akker J.T., Burm B.E., Ekhart P.F., Heuvelmans N., Holling T., Janson A.A., Platenburg G.J., Sipkens J.A. (2011). Systemic administration of PRO051 in Duchenne’s muscular dystrophy. N. Engl. J. Med..

[27] Goemans N., Mercuri E., Belousova E., Komaki H., Dubrovsky A., McDonald C.M., Kraus J.E., Lourbakos A., Lin Z., Campion G. (2018). A randomized placebo-controlled phase 3 trial of an antisense oligonucleotide, drisapersen, in Duchenne muscular dystrophy. Neuromuscul. Disord. NMD.

[28] Voit T., Topaloglu H., Straub V., Muntoni F., Deconinck N., Campion G., De Kimpe S.J., Eagle M., Guglieri M., Hood S. (2014). Safety and efficacy of drisapersen for the treatment of Duchenne muscular dystrophy (DEMAND II): An exploratory, randomised, placebo-controlled phase 2 study. Lancet. Neurol..

[29] McDonald C.M., Wong B., Flanigan K.M., Wilson R., de Kimpe S., Lourbakos A., Lin Z., Campion G., the Demand V study group (2018). Placebo-controlled Phase 2 Trial of Drisapersen for Duchenne Muscular Dystrophy. Ann. Clin. Transl. Neurol..

[30] Agrawal S. (1996). Antisense oligonucleotides: Towards clinical trials. Trends Biotechnol..

[31] Brown D.A., Kang S.H., Gryaznov S.M., DeDionisio L., Heidenreich O., Sullivan S., Xu X., Nerenberg M.I. (1994). Effect of phosphorothioate modification of oligodeoxynucleotides on specific protein binding. J. Biol. Chem..

[32] Weidner D.A., Valdez B.C., Henning D., Greenberg S., Busch H. (1995). Phosphorothioate oligonucleotides bind in a non sequence-specific manner to the nucleolar protein C23/nucleolin. FEBS Lett..

[33] Shen W., Liang X.H., Crooke S.T. (2014). Phosphorothioate oligonucleotides can displace NEAT1 RNA and form nuclear paraspeckle-like structures. Nucleic Acids Res..

[34] Liang X.H., Sun H., Shen W., Crooke S.T. (2015). Identification and characterization of intracellular proteins that bind oligonucleotides with phosphorothioate linkages. Nucleic Acids Res..

[35] Liang X.H., Shen W., Sun H., Kinberger G.A., Prakash T.P., Nichols J.G., Crooke S.T. (2016). Hsp90 protein interacts with phosphorothioate oligonucleotides containing hydrophobic 2′-modifications and enhances antisense activity. Nucleic Acids Res..

[36] Shen W., De Hoyos C.L., Migawa M.T., Vickers T.A., Sun H., Low A., Bell T.A., Rahdar M., Mukhopadhyay S., Hart C.E. (2019). Chemical modification of PS-ASO therapeutics reduces cellular protein-binding and improves the therapeutic index. Nat. Biotechnol..

[37] Goyenvalle A., Griffith G., Babbs A., El Andaloussi S., Ezzat K., Avril A., Dugovic B., Chaussenot R., Ferry A., Voit T. (2015). Functional correction in mouse models of muscular dystrophy using exon-skipping tricyclo-DNA oligomers. Nat. Med..

[38] Relizani K., Griffith G., Echevarria L., Zarrouki F., Facchinetti P., Vaillend C., Leumann C., Garcia L., Goyenvalle A. (2017). Efficacy and Safety Profile of Tricyclo-DNA Antisense Oligonucleotides in Duchenne Muscular Dystrophy Mouse Model. Mol. Ther. Nucleic Acids.

[39] Koshkin A.A., Singh S.K., Nielsen P., Rajwanshi V.K., Kumar R., Meldgaard M., Olsen C.E., Wengel J. (1998). LNA (Locked Nucleic Acids): Synthesis of the adenine, cytosine, guanine, 5-methylcytosine, thymine and uracil bicyclonucleoside monomers, oligomerisation, and unprecedented nucleic acid recognition. Tetrahedron.

[40] Deleavey G.F., Damha M.J. (2012). Designing chemically modified oligonucleotides for targeted gene silencing. Chem. Biol..

[41] Aartsma-Rus A., Kaman W.E., Bremmer-Bout M., Janson A.A., den Dunnen J.T., van Ommen G.J., van Deutekom J.C. (2004). Comparative analysis of antisense oligonucleotide analogs for targeted DMD exon 46 skipping in muscle cells. Gene Ther..

[42] Shimo T., Tachibana K., Saito K., Yoshida T., Tomita E., Waki R., Yamamoto T., Doi T., Inoue T., Kawakami J. (2014). Design and evaluation of locked nucleic acid-based splice-switching oligonucleotides in vitro. Nucleic Acids Res..

[43] Pires V.B., Simoes R., Mamchaoui K., Carvalho C., Carmo-Fonseca M. (2017). Short (16-mer) locked nucleic acid splice-switching oligonucleotides restore dystrophin production in Duchenne Muscular Dystrophy myotubes. PLoS ONE.

[44] Le B.T., Adams A.M., Fletcher S., Wilton S.D., Veedu R.N. (2017). Rational Design of Short Locked Nucleic Acid-Modified 2′-*O*-Methyl Antisense Oligonucleotides for Efficient Exon-Skipping In Vitro. Mol. Ther. Nucleic Acids.

[45] Christou M., Wengel J., Sokratous K., Kyriacou K., Nikolaou G., Phylactou L.A., Mastroyiannopoulos N.P. (2020). Systemic Evaluation of Chimeric LNA/2′-*O*-Methyl Steric Blockers for Myotonic Dystrophy Type 1 Therapy. Nucleic Acid Ther..

[46] Heemskerk H.A., de Winter C.L., de Kimpe S.J., van Kuik-Romeijn P., Heuvelmans N., Platenburg G.J., van Ommen G.J., van Deutekom J.C., Aartsma-Rus A. (2009). In vivo comparison of 2′-*O*-methyl phosphorothioate and morpholino antisense oligonucleotides for Duchenne muscular dystrophy exon skipping. J. Gene Med..

[47] Mann C.J., Honeyman K., McClorey G., Fletcher S., Wilton S.D. (2002). Improved antisense oligonucleotide induced exon skipping in the mdx mouse model of muscular dystrophy. J. Gene Med..

[48] Spitali P., Heemskerk H., Vossen R.H., Ferlini A., den Dunnen J.T., t Hoen P.A., Aartsma-Rus A. (2010). Accurate quantification of dystrophin mRNA and exon skipping levels in duchenne muscular dystrophy. Lab. Investig. J. Tech. Methods Pathol..

[49] Schnell F.J., Fletcher S., Johnsen R.S., Wilton S.D. (2019). Challenges of Interpreting Dystrophin Content by Western Blot. US Neurol..

[50] Townsend D., Yasuda S., Li S., Chamberlain J.S., Metzger J.M. (2008). Emergent dilated cardiomyopathy caused by targeted repair of dystrophic skeletal muscle. Mol. Ther. J. Am. Soc. Gene Ther..

[51] Malerba A., Boldrin L., Dickson G. (2011). Long-term systemic administration of unconjugated morpholino oligomers for therapeutic expression of dystrophin by exon skipping in skeletal muscle: Implications for cardiac muscle integrity. Nucleic Acid Ther..

[52] Jearawiriyapaisarn N., Moulton H.M., Sazani P., Kole R., Willis M.S. (2010). Long-term improvement in mdx cardiomyopathy after therapy with peptide-conjugated morpholino oligomers. Cardiovasc. Res..

[53] Betts C., Saleh A.F., Arzumanov A.A., Hammond S.M., Godfrey C., Coursindel T., Gait M.J., Wood M.J. (2012). Pip6-PMO, A New Generation of Peptide-oligonucleotide Conjugates With Improved Cardiac Exon Skipping Activity for DMD Treatment. Mol. Ther. Nucleic Acids.

[54] Gait M.J., Arzumanov A.A., McClorey G., Godfrey C., Betts C., Hammond S., Wood M.J.A. (2019). Cell-Penetrating Peptide Conjugates of Steric Blocking Oligonucleotides as Therapeutics for Neuromuscular Diseases from a Historical Perspective to Current Prospects of Treatment. Nucleic Acid Ther..

[55] Rosenblatt J.D., Lunt A.I., Parry D.J., Partridge T.A. (1995). Culturing satellite cells from living single muscle fiber explants. Vitr. Cell. Dev. Biol. Anim..

[56] Wisniewski J.R., Zougman A., Nagaraj N., Mann M. (2009). Universal sample preparation method for proteome analysis. Nat. Methods.

[57] Alelyunas Y.W., Wrona M.D., Mortishire-Smith R.J., Tomczyk N., Rainville P.D. Quantitation by High Resolution Mass Spectrometry: Using Target Enhancement and Tof-MRM to Achieve Femtogram-level On-column Sensitivity for Quantitation of Drugs in Human Plasma. https://www.waters.com/webassets/cms/library/docs/720005182en.pdf.

[58] MacLean B., Tomazela D.M., Shulman N., Chambers M., Finney G.L., Frewen B., Kern R., Tabb D.L., Liebler D.C., MacCoss M.J. (2010). Skyline: An open source document editor for creating and analyzing targeted proteomics experiments. Bioinformatics.

