# Supplementary material for: Intramuscular Evaluation of Chimeric Locked Nucleic Acid/2′OMethyl-Modified Antisense Oligonucleotides for Targeted Exon 23 Skipping in Mdx Mice
Source: Pharmaceuticals (Basel). 2021 Oct 30;14(11):1113. doi: 10.3390/ph14111113 (PMC8622172; doi:10.3390/ph14111113)
Supplement: Supplementary file 1 [file pharmaceuticals-14-01113-s001.zip › pharmaceuticals-1421922-supplementary.pdf]

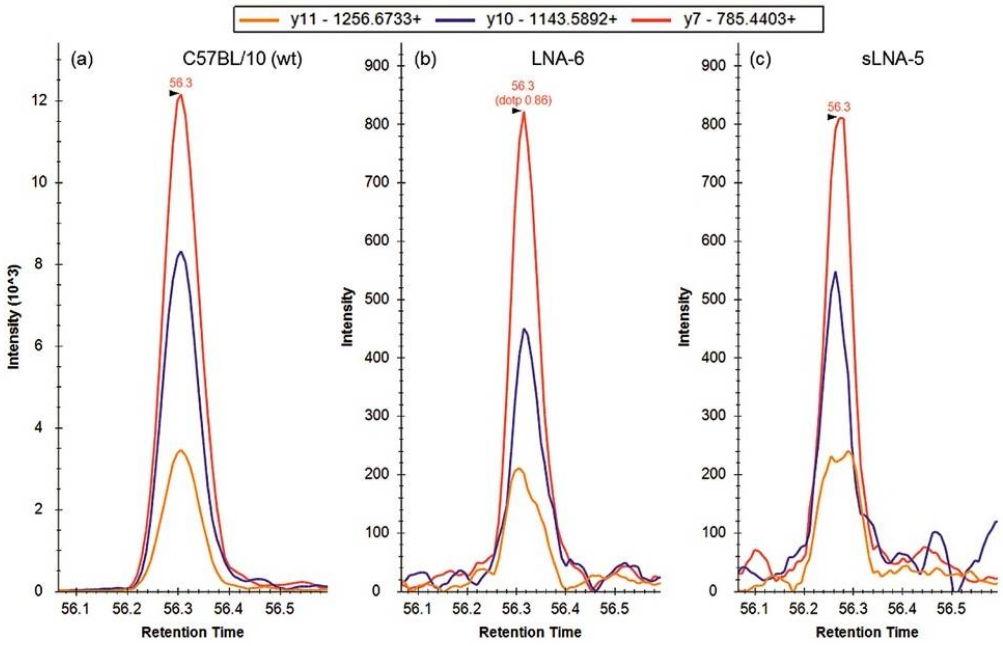

Figure S1. Representative HD-MRM chromatograms of selected transitions (product ions) of the IFLTEQPLEGLEK peptide (m/z 758.9165 Da), as measured in muscle protein extracts of a WT control and mdx mice treated with AONs LNA-6 and sLNA-5. All three transitions can be clearly observed, while the ratio of intensity among the different transitions is maintained in all three samples.

Table S1. UPLC Elution profile and LC parameters.

Time (min)	Flow Rate (µL/min)	% A	% B	LC System: nanoAcquity UPLC
0	0.300	99	1	Mobile phase A: 0.1% Formic acid in Water
4	0.300	99	1	
7	0.300	95	5	Mobile phase B: 0.1% Formic acid in Acetonitrile
15	0.300	95	5	
72	0.300	69	31	Column: Acquity M-Class 75 µm × 250 mm, 1.7 µm
75	0.300	10	90	
85	0.300	10	90	Column temperature: 45.0 °C
85.1	0.300	99	1	
100	0.300	99	1	Sample temperature: 6 °C

Table S2. HD-MRM transitions and MS parameters for dystrophin peptides.

Peptide	RT (min)	MRM Transition	Cone (volts)	CE (Volts)	Production
		758.916 > 785.440			y7 +
IFLTEQPLEGLEK	56.3	758.916 > 1143.673	30	20	y10 +
		758.916 > 1256.673			y11 +
		527.319 > 756.425			y6 +
LLAEELPLR	49.1	527.319 > 827.462	32	29	y7 +
		527.319 > 940.546			y8 +
